# Fibroblasts in the Tumor Microenvironment: Shield or Spear?

**DOI:** 10.3390/ijms19051532

**Published:** 2018-05-21

**Authors:** Twana Alkasalias, Lidia Moyano-Galceran, Marie Arsenian-Henriksson, Kaisa Lehti

**Affiliations:** 1 Department of Microbiology, Tumor and Cell Biology (MTC), Karolinska Institutet, Biomedicum, Solnavägen 9, SE-17177 Stockholm, Sweden; lidia.moyano.galceran@ki.se (L.M.-G.); Marie.Arsenian.Henriksson@ki.se (M.A.-H.); 2 Department of Biology, College of Science, Salahaddin University, Irbil 44002, Kurdistan-Iraq; 3 Research Programs Unit, Genome-Scale Biology and Medicum, University of Helsinki, and Helsinki University Hospital, P.O. Box 63, FI-00014 Helsinki, Finland

**Keywords:** normal fibroblasts, cancer-associated fibroblasts, neighbor suppression, cancer, desmoplasia, therapy

## Abstract

Tumorigenesis is a complex process involving dynamic interactions between malignant cells and their surrounding stroma, including both the cellular and acellular components. Within the stroma, fibroblasts represent not only a predominant cell type, but also a major source of the acellular tissue microenvironment comprising the extracellular matrix (ECM) and soluble factors. Normal fibroblasts can exert diverse suppressive functions against cancer initiating and metastatic cells via direct cell-cell contact, paracrine signaling by soluble factors, and ECM integrity. The loss of such suppressive functions is an inherent step in tumor progression. A tumor cell-induced switch of normal fibroblasts into cancer-associated fibroblasts (CAFs), in turn, triggers a range of pro-tumorigenic signals accompanied by distraction of the normal tissue architecture, thus creating an optimal niche for cancer cells to grow extensively. To further support tumor progression and metastasis, CAFs secrete factors such as ECM remodeling enzymes that further modify the tumor microenvironment in combination with the altered adhesive forces and cell-cell interactions. These paradoxical tumor suppressive and promoting actions of fibroblasts are the focus of this review, highlighting the heterogenic molecular properties of both normal and cancer-associated fibroblasts, as well as their main mechanisms of action, including the emerging impact on immunomodulation and different therapy responses.

## 1. Introduction

**Figure 1:**
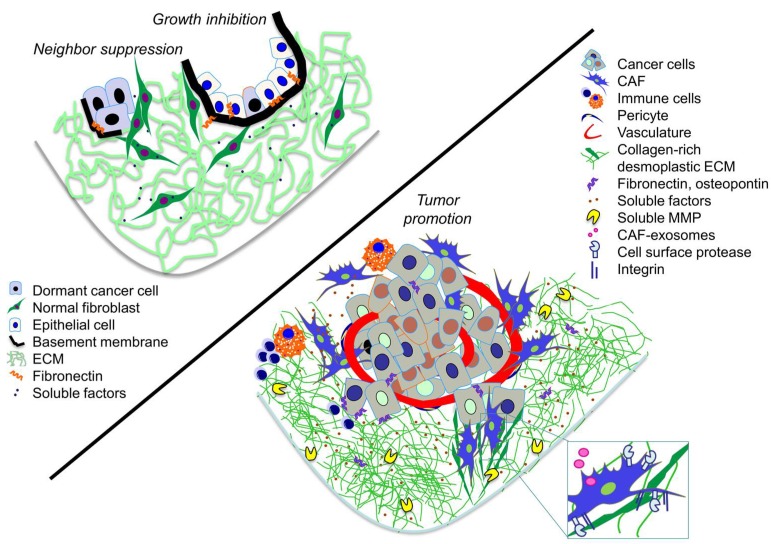
The dual action of fibroblast in the TME. Illustrative scheme showing the interactions and products of the anti-tumorigenic normal fibroblasts (upper-left) and the pro-tumorigenic cancer associated fibroblasts (CAFs) (lower-right).

The concept of the tumor microenvironment (TME) encompasses the stromal components, which surround the cancer cells and have a major impact on the processes of tumorigenesis. By contributing to the majority of the hallmark capabilities and characteristics of cancer cells, ranging from sustained proliferative signaling, resistance to cell death, genome instability, induction of angiogenesis and tumor-promoted inflammation, evasion of both growth suppressors and immune destruction to reprogrammed energy metabolism, as well as activation of invasion and metastasis, TME drives the evolution of a heterogeneous disease [1]. The TME is composed of cells, such as fibroblasts, endothelial cells, pericytes, macrophages, lymphocytes, and other immune cells, as well as an acellular compartment; the extracellular matrix (ECM) and associated soluble factors, all of which can differ according to the type, stage, and location of the cancer. The stromal cells interact with each other and with the cancer cells in a dynamic and context dependent manner [2]. The outcome of such tumor-stroma crosstalk is either issuing alliances to promote carcinogenesis, or negatively regulating cancer cell growth. While the normal stroma confers anti-tumorigenic activities to restrict the tumor initiation and growth, some cancer cells can tolerate the suppression and, in turn, start to reprogram and remodel the TME into one conferring cancer-supporting functions [3]. Such a transition, achieved by active cell recruitment and the progressive changes of the stromal cells from normal to a tumor-associated phenotype, is a critical driver of tumor evolution. Herein, we highlight the paradoxical functions of fibroblasts (Figure 1), which represent both a major cellular component and a source of ECM in the TME, to regulate cancer growth and progression in a context-dependent manner.

## 2. Normal Fibroblasts: The Anti-Tumorigenic Response

### 2.1. The Function of Normal Fibroblasts

Fibroblasts constitute one of the most abundant cell types in the stroma. These cells produce and reorganize various ECM proteins, which are essential elements in normal tissue homeostasis and function [4]. Fibroblasts also affect the recruitment of immune cells via, e.g., Toll-like receptors, production of inflammatory mediators, and sensitizing the immune cells to bacterial lipopolysaccharide [5]. According to their anatomical site of origin, as well as the host stromal tissue type and state, fibroblasts can display heterogeneous phenotypes by exhibiting different transcriptional programs collectively controlled by epigenetic modifications and local signals [6]. Similar to the fibroblast tissue specificity, the configuration of their surrounding ECM varies according to the tissue localization and type. Such diversity, as well as the context-dependent expression and activities of the adhesion molecules and ECM remodeling enzymes, provides a framework for the tissue specific resident cells to negotiate with and navigate through the adjacent tissue [7]. Examples of the ECM proteins produced by fibroblasts include fibrillar collagens (e.g., type I, III and V), proteoglycans, fibronectin, glycosaminoglycans, as well as other glycoproteins and fibrils, which all together, configure a three-dimensional network and generate osmotic-active scaffolds in the stromal interstitial tissues [4,8].

Fibroblasts also participate in the formation of sub-epithelial/endothelial basement membranes by synthesizing and secreting laminins and collagen IV, as well as other basement membrane-associated proteins [9]. Depending on the tissue type and localization, the fibroblasts can interact and communicate with the surrounding ECM through membrane protein complexes, including adhesion and signaling molecules. As a result, and depending on other type of stimuli received, the fibroblasts can initiate responses to synthesize and/or degrade particular ECM structures and molecules [10]. Different cell-surface adhesion receptors such as integrins, syndecans, and cadherins are expressed by fibroblasts and function as mediators of interactions with the ECM and other cells. Particularly, the collagen and fibronectin binding integrins have been found to be essential for remodeling the surrounding matrix [4]. Other mechanisms of ECM remodeling occur via the secretion of matrix degrading and crosslinking enzymes [11]. The expression of these ECM modulators is regulated by various pro-inflammatory cytokines and growth factors, such as interleukin (IL)-1α, IL-1β, and the other IL family members, fibroblast growth factors (FGFs), transforming growth factor β (TGFβ) family members, and platelet-derived growth factors (PDGFs) [11,12]. The main families of proteolytic enzymes include matrix metalloproteinases (MMPs), cathepsins, as well as urokinase-plasminogen system proteins and e.g., type II transmembrane serine proteases [13]. The protease activities are opposed by inhibitors, such as tissue inhibitors of metalloproteinases (TIMPs) and plasminogen activator inhibitor, as well as systemic inhibitors, like α2-macroglobulin, to collectively control tissue maintenance and repair [13].

### 2.2. The Neighbor Suppression Phenomenon

Is accumulation of mutations enough to transform a normal cell into a cancer cell that will consecutively develop a malignant tumor? If this was the case, cancer incidence would be expected to be even higher, considering the spontaneous mutations arising frequently in human cells even in the presence of effective DNA proofreading and repair mechanisms [14]. However, the majority of people remain cancer-free throughout their lifespan; therefore, resistance mechanisms can also be expected to operate in preventing the transformed putative cancer initiating cells from developing into a malignant tumor [15]. One such surveillance mechanism against cancer development and progression is driven by immune cells killing the defective mutant cells, which is especially effective in viral-induced carcinogenesis [16]. Other prominent mechanisms have been found to occur via normal fibroblasts and the tumor-suppressive ECM produced by them. This is of particular interest when considering the emerging functional plasticity of tumor cells, including their critical capabilities to modulate and/or avoid the immune surveillance [17,18,19].

Upon contact, normal fibroblasts can inhibit the growth of adjacent abnormal or transformed cells via “neighbor suppression” [18]. Michael Stoker and co-workers first discovered this phenomenon when they found that mouse fibroblasts, upon contact, inhibited the growth of polyoma virus-transformed cells in vitro [20]. Since then, several reports have described the suppressive effect of fibroblasts against cancer cell growth by variable mechanisms, including gap junction-dependent and -independent growth inhibition [21,22,23]. Moreover, soluble factors, such as TGFβ, tumor necrosis factor α (TNFα), and IL-6, secreted by the fibroblasts can mediate contact-independent tumor suppression in a paracrine manner [24,25,26]. Our results indicate that the inhibition of cancer cell proliferation and motility by fibroblasts is both contact- and soluble factor-dependent [27]. The cell-cell contacts can not only directly mediate cell growth suppression, but also maintain secretion of a range of soluble factors, which further potentiate cancer cell growth inhibition [27]. Coincidentally, several signaling cascades become deregulated in fibroblasts, including the focal adhesion, TNFα, and Ras homolog gene family, member A (RhoA) pathways [28].

The diversity in the putative mechanisms underlying neighbor suppression raises questions about the effects of fibroblast heterogeneity, plasticity, and tissue specificity in this phenomenon. George Klein and colleagues have shown that different types of fibroblasts can inhibit cancer cell proliferation with varying efficiencies in a manner dependent on the fibroblast site of origin and the age of the donor; human skin and pediatric fibroblasts being more effective at suppressing cancer cell proliferation than internal organ and adult fibroblasts [29]. Moreover, mouse dermal fibroblasts have been shown to reduce p16 and cyclin D1 levels in melanoma cells, thus inhibiting tumor development in vivo [30]. In a mouse pancreatic ductal adenocarcinoma (PDAC) model, a more aggressive tumor phenotype results from stromal reduction by sonic hedgehog gene deletion [31]. In a related mouse model with the depletion of α-smooth muscle actin (αSMA)-positive fibroblasts, highly-invasive tumors develop with characteristics of enhanced epithelial to mesenchymal transition (EMT), hypoxia, and stem cell-like properties, in association with reduced animal survival [32]. However, several questions remain to be addressed regarding the mechanisms and relevance of the neighbor suppression phenomenon mediated by the stromal cells against the neoplastic epithelium. Further comprehensive characterization of the context-dependent and tissue specific fibroblast properties and subsets that suppress tumor growth and development is required to increase the understanding of this phenomenon.

### 2.3. Tissue Architecture

Fibroblasts support and maintain the architecture of tissues and organs, thereby providing them with appropriate microenvironmental conditions to perform normal tissue functions [33]. Evidence exists to indicate that destroying these structures is a prerequisite for malignant tumor development and growth. More than three decades ago, Mina Bissell and co-workers reported that the destruction of the normal tissue architecture by wound injury enables tumor development in Rous sarcoma virus-carrying chickens [34]. On the other hand, mice develop normally after injection of mouse teratocarcinoma cells into albino mouse blastocytes; however, these mice carry small cancer cell colonies in the normal organs during their entire lifespan [35]. Tumor foci can also be frequently found in humans when the tissues of cancer-free individuals are carefully examined [36], which is consistent with the consideration that the normal microenvironment can restrain the cancer cells from developing into an ample malignant growth via neighbor suppression-dependent tumor dormancy. An example of experimental evidence for cancer growth prevention by fibroblast-maintained tissue architecture is provided by the naked mole rat model [37]. In these cancer resistant rodents, the fibroblasts secrete high molecular weight hyaluronan which protects the animal from developing cancer by early contact inhibition and tissue homeostasis dependent on the CD44-Neurofibromin 2 pathway [37].

Recurrence of cancer over ten years after detection and removal of the primary tumor is, in turn, an example of the awakening of dormant cancer cells at the metastatic site. It has been suggested that destruction of microenvironmental architecture is one key enabling mechanism for this process [38]. Therefore, the distraction of the ability of fibroblasts to reorganize ECM via manipulating their mechanical and adhesive properties can confer a pro-tumorigenic microenvironment. RhoA is a master regulator of cell shape, adhesiveness, contractile behavior, and the configuration of focal adhesions [39]. Our studies showed that knockout of the RhoA gene in fibroblasts disturbs cytoskeletal organization, increases cellular stiffness, and decreases cellular contractility [40], which all are important properties required to maintain normal connective tissue structure and function [39,40]. As a result, the fibroblasts lose their tumor suppressive function and, rather, provide a growth stimulatory niche via induction of a cancer propagating phenotype in 3D-collagen culture and xenograft tumors in vivo [40]. Altogether, these results indicate that maintaining tissue integrity is crucial to prevent tumor development.

Based on these observations, the following question arises: is it possible to phenotypically normalize cancer cells and, thus, halt tumor growth via reconstituting the stromal integrity? There are findings that support this hypothesis, such as the detection of the same mutation in both obvious tumor cells and in adjacent, more normal-like cells [41]. This suggests that the malignant cells can be enforced in the perspective of phenotypic normalization depending on their microenvironment. Consistently, when attempting to reconstruct a human mammary epithelial tissue in mice, Weinberg and colleagues found that normal fibroblasts are responsible for the configuration of the normal epithelial phenotype. Furthermore, patient epithelial cells injected in a humanized cancer associated microenvironment develop into a cancer that resembles human ductal carcinoma, and this cancer cell phenotype can be normalized by co-injection of normal fibroblasts with the epithelial cells [42]. Therefore, the identification of mechanisms for reconstituting the normal stromal architecture in cancer can open new possibilities and treatment modalities for better cancer prevention and treatment.

## 3. Fibroblasts Changing Identity: The Switch from Suppressors to Tumor Promoters

Malignancies arise when transformed cells overcome the normal cellular surveillance. Eventually, the structure and function of the TME will change, switching from the initial anti-tumorigenic activities and properties to those supporting and protecting tumor cells [43]. The gain of such tumor supportive functions occurs gradually and concurrently with the loss of tumor inhibition. Furthermore, the disturbance of stromal architecture can lead to the accumulation of tissue damage, which in turn initiates different signaling cascades that alter cancer cell proliferation, invasion, and other functions [10]. 

### 3.1. The Activation of Fibroblasts by Tumor Cells

Normal adult fibroblasts in physiological conditions display a quiescent phenotype, which from a molecular perspective still remains incompletely characterized [44]. However, upon various stimuli, the physiological status of the fibroblasts is known to change, allowing them to become activated and display inexhaustible protein synthetic activity and contractile functions [45,46]. When compared to quiescent fibroblasts, the activated cells are more migratory and vulnerable to epigenetic modifications, enabling their function as precursors for different cell types.

In cancer, fibroblasts are continuously exposed to different stimuli, which promote unique features, such as excessive and specific secretory and ECM remodeling phenotypes. In addition, the cancer-associated fibroblasts (CAFs) can acquire an increased autocrine signaling ability and proliferative efficiency [47]. Generally, the switch from normal fibroblasts to tumor invasion and growth-promoting CAFs is considered to require epigenetic modifications. For example, leukemia inhibitory factor induces an epigenetic switch in fibroblasts that leads to the acquisition of the CAF phenotype [48]. Moreover, normal fibroblasts can be converted into CAFs by the action of miRNAs contained in tumor-derived exosomes and microvesicles; for example, miR-214 in ovarian cancer and miR-155 in pancreatic cancer can induce this conversion [49,50]. Independent of the switch mechanism, the induction of immunomodulatory CAF functions leads to the massive production of cytokines and chemokines, including PDGF, vascular endothelial growth factor A (VEGFA), prostaglandin E2, IL-6, TNF, nuclear factor kappa-light-chain-enhancer of activated B cells (NF-κB), IL-8, hepatocyte growth factor (HGF), and stromal cell-derived factor 1 (SDF1, also called CXCL12) [47,51]. Moreover, the specific ECM remodeling ability of CAFs is attributed to the production of multiple MMPs, such as MMP1, MMP2, MMP3, MMP9, MMP13, MMP14, and TIMPs [52,53,54,55].

Cancer-associated fibroblasts can be identified both in vitro and in vivo through a panel of marker proteins/genes such as PDGFRα/β, αSMA, fibroblast-associated protein (FAP), and fibroblast-specific protein 1 (FSP1), whereas absolute markers for the identification of quiescent fibroblasts are still under debate [56,57]. The broadly used marker for both normal and activated fibroblasts is FSP1, also called S100A4. However, this protein is also expressed in several types of immune cells and certain cancer cells [58,59,60,61].

**Figure 2:**
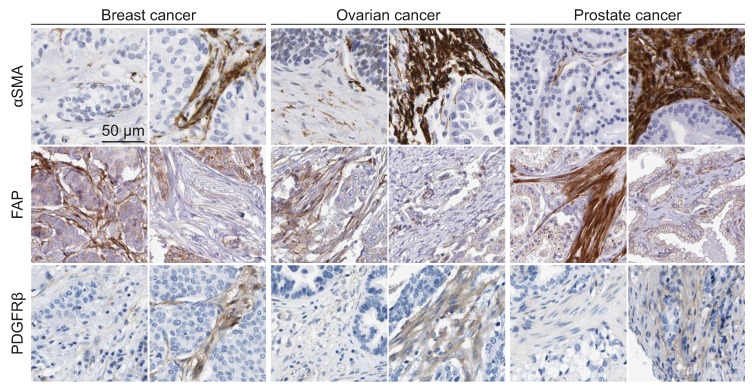
The heterogeneity of CAFs. Immunohistochemical stainings of aSMA (CAB000002), FAP (HPA059739,) and PDGFRβ (CAB018144) of tumor sections from breast (duct carcinoma), ovarian (cystadenocarcinoma, serous), and prostate (adenocarcinoma, high grade) cancers. These stainings show the heterogeneity in CAF markers not only between cancers, but also between patients with the same cancer type. Images were obtained from the Human Protein Atlas (Available from www.proteinatlas.org, [65]).

Recently, different studies have defined distinct CAF signatures. For example, twelve new CAF markers (ARHGAP26, ARHGAP31, AZI2, BHLHE40, DLG1, EGLN1, ITCH, PKM2, PLOD2, RAB31, ROCK2, and RNF19A) have been identified by the analysis of more than 2500 proteins using the Protein Atlas database [62]. This signature identifies CAFs of five different cancers, including lung, colorectal, breast, basal cell, and squamous cell carcinoma. In a colon cancer study, a quantitative proteomics analysis has identified a new CAF signature assembled by four markers (CDH11, FSTL1, LTBP2, and OLFML3) [63]. Altogether these observations indicate that CAFs represent highly heterogeneous cell populations, displaying defined gene signatures and protein expression patterns depending on the type of cancer, and also varying among patients with the same cancer type (Figure 2). Since those CAF signatures can cluster the patients into high and low risk groups, improved understanding of such variability can prove useful for the development of prognostic factors [64]. Altogether, the identification of specific CAF subsets in different cancers will enrich our knowledge in the field, and open new avenues for the development of novel cancer diagnosis and treatment strategies.

### 3.2. The Origin of CAFs

The tissue-resident fibroblasts are often considered as the main source of fibroblast-like cells [66]; however, their low proliferative potential challenges the model of local fibroblast activation [33,67]. Epithelial cells are another putative source of fibroblast-like cells, since they can shift into fibroblast-like phenotype through EMT in inflammation and cancer [68]. During EMT, the epithelial cell loses the cell-cell junctions and polarity, and experiences cytoskeletal reorganization and morphological changes that provide the cell with a mesenchymal phenotype and invasive capability [69]. Mesenchymal stromal cells have also been considered as precursors of fibroblast-like cells, which conclusion is supported by genetic tracing results from two models of bone marrow fibrosis [70]. Similar to normal fibrosis, fluorescent-cell tracing results in murine models of gastric and pancreatic cancer have indicated that mesenchymal stem cells from bone marrow can be recruited to the tumor niche and converted into CAFs in response to TGFβ and SDF1α signaling [71,72]. In melanoma and pancreatic cancer models, the endothelial cells are subjected to an endothelial-to-mesenchymal transition (EndMT) mediated by autocrine and paracrine TGFβ signaling that turns them into precursors for CAFs [73].

**Figure 3:**
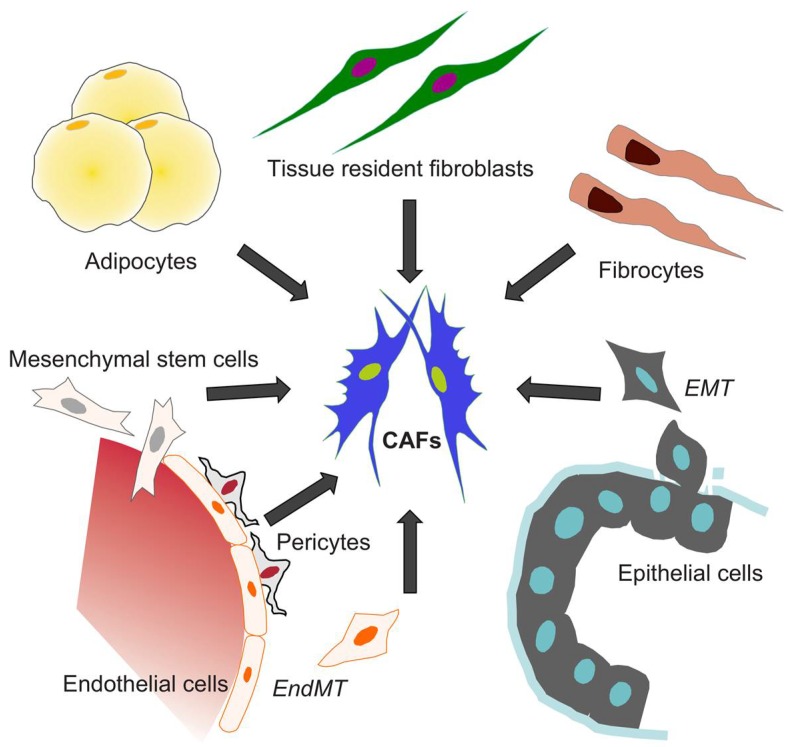
The origin of CAFs. Different cell types can transdifferentiate and represent the source of CAFs, including: tissue resident fibroblasts, fibrocytes, epithelial cells (through EMT), adipocytes, mesenchymal stem cells, pericytes, and endothelial progenitor cells (through EndMT).

Another possible source for cancer-associated accumulation of fibroblasts is fibrocytes, which constitute less than 0.5% of non-erythrocytic cells in the blood [74]. These cells can induce tissue remodeling upon entry into the site of injury by differentiating into fibroblasts in response to TGFβ and other cytokines [75,76]. Since both fibroblasts and adipocytes are of the same mesenchymal lineage, the adipocytes have also been suggested to represent a source of CAFs [77]. Moreover, adipose tissue mesenchymal cells can be converted to fibroblast-like cells that induce the growth of human pancreatic cancer cells in BALB/cAJcl-nu/nu mice [78]. The heterogeneity of CAFs, highlighted by the specific expression patterns of markers for cell identity and differentiation, as well as the mechanistic diversity in supporting carcinogenesis, is consistent with the notion that CAFs can originate and be recruited to the tumor from different tissues and cell types (Figure 3).

### 3.3. Functions of CAFs in Cancer Initiation

Multiple observations from in vitro and in vivo studies highlight the contribution of CAFs in the process of cancer initiation. CAFs isolated from prostate cancer patients can induce epithelial cell transformation and immortalization, as well as shift the non-tumorigenic features of the epithelial cells into highly tumorigenic ones [79]. Different experimental strategies, such as gene modification, overexpression, and deletion, have been used to demonstrate the contribution of stromal fibroblasts (and possibly other heterogeneous fibroblast marker-expressing cells) in tumor development. For instance, fibroblasts overexpressing Wingless-type MMTV integration site family member 1 (Wnt1) can transform mammary epithelial cells isolated from C57BL/6 mice [80]. Moreover, TGFβ receptor II gene knock-out in FSP1-positive cells promotes prostate intraepithelial neoplasia and fore-stomach squamous cell carcinoma [81]. In the FSP1 null mouse model, the mice instead display significantly delayed and decreased tumor initiation upon injection of highly metastatic mouse mammary carcinoma cells, whereas the co-injection with FSP1-positive fibroblasts restores tumor development and enhances metastasis [82]. Phosphatase and tensin homolog (PTEN) inactivation in fibroblasts significantly enhances the malignant transformation, initiation, and growth of mammary adenocarcinoma in mice, coinciding with immune cell infiltration and substantially increased ECM remodeling [83]. Moreover, the transcriptome analysis of PTEN-inactivated fibroblasts shows a strong correlation with breast CAFs in human patients [83]. The deletion of liver kinase B1 in stromal fibroblasts has also been found to induce gastrointestinal cancers in a mouse model through an effect associated with increased IL-11 production by fibroblasts coupled to activation of the Janus kinases/signal transducer and activation of the transcription proteins (JAK/STAT3) pathway in tumor cells [84].

The above results suggest that the switch of normal stroma into CAF-containing TME is one of the fundamental steps controlling tumor development. However, due to the difficulty in defining the threshold of cancer onset, the impact of fibroblasts on tumor initiation is under debate. The concept of “the egg and the chicken” is applicable with regards to who comes first. Do the cancer cells recruit fibroblasts to help in malignant growth and dissemination, or are the activated fibroblasts required early on to support the malignancy of the hyperplastic epithelium? The validity of such questions, in addition to the limited specificities of fibroblast lineage markers, reflects the challenges in creating clinically relevant experimental models to systematically follow and study the regulation of cancer initiation.

### 3.4. Regulation of Cancer Growth by CAFs

The cancer promoting CAF functions have been studied using various mouse and human cell experimental models. CAFs, but not normal fibroblasts, can induce tumor growth from hyperplastic prostate cells, whereas the same fibroblasts fail in inducing the growth of normal prostate epithelial cells [85]. This suggests that CAFs are not sufficient to induce tumor initiation, but instead promote the progression of an already initiated growth. To boost tumor development, CAFs can induce paracrine activities. C-X-C motif chemokine 12 secreted by CAFs enhances tumor growth by interacting with C-X-C chemokine receptor type 4 (CXCR4), thus inducing downstream signaling cascades, as well as cancer cell proliferation and motility in models of breast cancer [86], endometrial cancer [87], adenocarcinoma of the esophagogastric junction [88] and melanoma [89]. Moreover, CXCL14 autocrine signaling, which is dependent on the activation of nitric oxide synthase 1 in CAFs, enhances tumor growth in a prostate cancer model [90]. As shown in endometrial cancer, IL-6 secreted by CAFs stimulates cancer cell proliferation via the STAT3/c-MYC signaling pathway [91]. In a melanoma model, fibroblasts lacking pigment epithelium-derived factor can induce cancer cell growth both in vitro and in vivo, as the tumor stimulatory fibroblasts exhibit high expression of IL-8, plasminogen activator inhibitor-2, and hyaluronan synthase-2 [92]. Altogether, the pro-inflammatory cytokines and chemokines secreted by CAFs are, therefore, important for tumor growth and progression.

### 3.5. Functions of CAFs in Cancer Metastasis: From Initial Invasion to Tissue Colonization

The induction of cancer invasion across the epithelial and endothelial basement membranes typically involves coordinated adhesive and proteolytic activities altered at the cancer cell surface by invasion/EMT-inducing signals [93,94,95]. However, basement membrane invasion can also be achieved by CAFs or cancer cells pulling and stretching their plasma membranes [96,97]. This creates gaps in the basement membrane that allow the cancer cells to invade even without proteolytic MMP activity [96]. At the primary tumors, CAFs also act as guides for stromal dissemination by generating ECM tracks that pave the way for collective invasion of the cancer cells that have not undergone a full EMT, and thereby stay together by E-cadherin-mediated or possibly also other types of cell-cell adhesion [98,99]. Further support for the CAF-directed cancer invasion is provided by a zebrafish model, where prostate and colorectal cancer-derived fibroblasts induce metastasis during early primary cancer growth [100]. Most of these metastatic cells travel in tight association with CAFs. The CAF-directed cancer invasion to stroma can utilize matrix-degrading proteases at the surface of the leader fibroblasts [101], although the invasive cancer cells can also express the proteases, particularly MMP14, or use pre-existing tracks in tissues like dermis, whereby proteolysis is not needed in the leading front [97].

Broader TME alterations and ECM degradation by CAF-secreted MMPs also occur upon the metastatic processes. Examples of such tumor-promoting proteolytic mechanisms include CAF-mediated expression and activation of e.g., MMP1, MMP2, MMP3, and MMP9, which can disrupt tissue polarity and architecture, as well as enhance the abilities of cancer cells to suppress E-cadherin-mediated adhesion and navigate the stromal ECM constrains [102,103,104,105,106,107]. Unexpectedly, overexpression of the MMP inhibitor TIMP1 in CAFs has also been observed to support prostate and colon cancer progression in vivo [108]. However, depletion of all four members of the TIMP family in fibroblasts enhances breast cancer cell motility and cancer stem cell-like properties [108]. Such complete TIMP inactivation is sufficient for CAF activation, and subsequent secretion of exosomes rich in MMPs and ECM proteins. On the other hand, CAF-derived exosomes have been found to enhance migration and invasion of gastric cancer cells by inducing MMP2 expression in the cancer cells [109]. Exosomes containing a disintegrin and metalloproteinase 10 in turn activate the Notch signaling and RhoA in breast cancer cells, thus driving their activity and stem cell properties [110]. Inactivation of both Notch effector CSL and p53 have also been found to stimulate CAFs and cancer cell expansion [111].

In addition, CAFs secrete a range of cytokines, chemokines, and growth factors that promote cancer cell invasion and metastasis. For instance, IL-6 secreted by CAFs can activate the JAK2/STAT3 pathway in gastric cancer cells, thus boosting their migration and the ability to undergo EMT [112]. The inhibition of this paracrine signaling in either CAFs or cancer cells reduces the metastasis to the peritoneum [112]. These results coupled to the increased cancer growth in response to the JAK/STAT3 pathway activation by CAF-secreted IL-11 [84], highlight the central role of the paracrine signals via CAF-derived interleukins and JAK/STAT3 pathway in cancer cells controlling growth and motility.

High expression of the serum response factor in stromal fibroblasts induces cancer cell metastasis by CXCL12/CXCR4 signaling [113]. CXCL12 producing fibroblasts boost CXCL6 secretion in colon cancer cells, which consequently exhibit highly invasive and metastatic activities [114]. CXCL12 secreted by CAFs may also induce EMT, as has been reported for oral squamous cell carcinoma and breast cancer models [115,116]. The paracrine signals along with the ECM remodeling action of CAFs are, therefore, critically involved in cancer propagation. On the other hand, CAF-derived exosomes promote lung cancer cell invasion and metastasis by stimulating Wnt-regulation of planar cell polarity autocrine signaling in the cancer cells [117]. Exosomes holding miR-45 are secreted by in vitro activated fibroblasts, and can also be detected in the serum of esophageal squamous cell carcinoma patients [118]. These exosomes have been shown to induce cancer cell growth and migration [118].

Ultimately, cancer cells form metastatic tumors by tissue colonization of a distant organ. To achieve this, they may prime the target tissue in advance by recruiting stromal cells at the pre-metastatic site [119,120]. Infiltrating mammary cancer stem cells can prime and recruit lung fibroblasts to overexpress periostin, which stimulates Wnt signaling in cancer cells and enhances their colonization efficiency [121]. In PDAC metastasized to liver, resident hepatic stellate cells can be activated into periostin-secreting myofibroblasts through granulin secreted by tumor-associated macrophages (TAMs) [122]. At the metastatic niche, FSP1-positive cells have also been found to enhance cancer cell metastasis via VEGFA secretion, and depletion of these cells significantly reduces the metastatic colonization, while primary tumor growth remains unaffected [123]. Consistently, CAFs expressing connective tissue growth factor increase the micro-vessel density and tumor growth activity in a prostate cancer xenograft model [124]. Cancer-associated fibroblasts may, thus, further induce the angiogenic switch and formation of new vasculature in the metastatic TME.

### 3.6. The Desmoplastic Growth: CAFs and ECM

Desmoplasia is the reaction that leads to the accumulation of stromal components around a tumor, and is associated with poor clinical outcomes in cancer patients [125,126]. This process is characterized by high activity of CAFs that produce collagen-rich ECM and, together with the immune cells, constitute the majority of the desmoplastic growth [127,128]. In PDAC for instance, the accumulation of thick desmoplastic stroma that also contains high amounts of hyaluronan, promotes tumor growth in mice and correlates significantly with poor prognosis in patients [129].

In addition to altered biochemical signals provided by the desmoplastic TME, the production and re-assembly of ECM, including linear collagen structures, will change the physical properties and biomechanical activity of the microenvironment. Desmoplastic reaction induces tumor stiffness via processes closely attributed to the over-activation of lysyl oxidase (LOX), an enzyme that crosslinks collagen and other ECM components [130]. The increased tumor stiffness promotes integrin-based focal adhesion assembly and increases the formation of adhesion complexes in both cancer and stromal cells, thus creating an increasingly pro-tumorigenic microenvironment [130]. Increased integrin activity in CAFs transduces mechanical forces that further change the orientation of collagen and fibronectin fibers, promoting cancer growth and invasion. For instance, integrin α11, which is expressed together with αSMA in CAFs, induces stiffness of fibrillary collagen and promotes tumor growth and metastatic potential in non-small cell lung carcinoma [131]. In general, the generation of physical forces and stiffness-dependent cytoskeletal rearrangements are tightly linked to the dysregulation of Yes-associated protein 1 (YAP1) transcriptional co-activator in fibroblasts and cancer cells, which leads to transcriptional programs to further potentiate CAF activation and cancer cell growth [132]. On the other hand, increased α5β1 and α5β3 integrin activity in CAFs results in fibronectin alignment [133,134]. In combination with ECM degradative activities, this type of dynamic tumor tissue remodeling enables aggressive growth and invasion of the adjacent cancer cells [101,133,134].

In addition to integrins, the fibrillar collagen receptors in CAFs include the discoidin domain receptors DDR1/2, which can efficiently trigger intracellular signals through their tyrosine kinase activity [135]. In a metastatic breast cancer model, DDR2 has been found to be critical for tumor-associated ECM production and remodeling, and its depletion changes the ECM structure and composition to resemble an ECM produced by normal mammary fibroblasts [136]. The protease activity of CAFs is also induced in the increasingly collagen-rich TME to further modify the ECM configuration, thus providing favorable conditions for cancer growth and invasion, as well as the crosstalk between cancer cells and the ECM [137]. This type of dynamic ECM communication induces collective cancer cell invasion of colon cancer patient-derived organoids in collagen I rich microenvironment [137].

In addition to the remodeling of fibrillar ECM structures, CAFs secrete matricellular proteins, which serve as a link between the stromal ECM and the cancer cells, and alter variable cancer cell signaling cascades, thus enhancing invasion and metastasis [138]. For instance, tenascin C promotes Notch and Wnt signaling, inducing breast cancer cell metastasis to the lungs in mice and positively correlating with aggressiveness and poor survival in breast cancer patients [139,140]. This signaling link between ECM and cancer cells requires the expression of receptors on the cancer cells that bind to the matricellular proteins secreted by CAFs. As an example, osteopontin, which can be secreted by CAFs [141], is one type of matricellular protein that binds to integrins and the cell-surface proteoglycan CD44 on cancer cells, thus boosting their proliferation, survival, and invasion abilities [142]. Osteopontin-producing senescent fibroblasts enhance the pre-neoplastic growth of epithelial cells in vitro and in vivo through the activation of the mitogen-activated protein kinase (MAPK) pathway [143,144]. Therefore, modulating the ECM component biosynthesis, ECM reorganization and crosslinking of ECM-adhesive molecules may offer possibilities to reprogram the microenvironment to become tumor suppressive, thus helping to halt the tumor growth. Alternatively, blocking specific cancer cell surface adhesion receptor-ECM interactions may offer another possibility to hinder cancer cell invasion and metastasis.

### 3.7. Immune Modulation by CAFs

Cancer-associated fibroblasts persistently receive and/or respond to stimuli, which drive the dynamic evolution of their secretome during the different stages of tumorigenesis. As a result, they can affect other cells in the TME, in particular, the immune cells. Current evidence defines CAFs mainly as immunosuppressive agents [145].

For instance, IL-6 produced by CAFs restricts the maturation of dendritic cells and redirects monocytes towards macrophage differentiation [146,147]. Additionally, CAFs producing CXCL12 and C-C motif chemokine ligand 2 can recruit macrophages into the TME and support their differentiation into the TAM-2-activated phenotype [148]. Myeloid derived suppressor cells can also be recruited by fibroblast-secreted chemokines and have the potency to induce angiogenesis, participate in recruitment of regulatory T cells, as well as to inhibit the activity of both natural killer (NK) and T cells in the TME [149]. CAF-mediated immune suppression of the TME promotes tumor development and metastasis in murine breast cancer. In this case, depletion of CAFs via targeting FAP-positive cells results in recruitment of cytotoxic T cells and dendritic cells, in conjunction with decreased recruitment of pro-tumorigenic TAMs and a reduced angiogenic switch [150]. Similarly, upon depletion of FAP in fibroblasts, only 2% of the injected tumor cells can develop into a solid tumor and the anti-tumorigenic effect is mediated through interferon-γ and TNFα, besides the recruitment of CD8+ T cells into the TME [151]. Notably, TGFβ signaling in CAFs induces an immune-exclusion phenotype in the tumor, which can be reverted by targeting TGFβ in the TME. The inhibition of this TGFβ signaling facilitates T cell penetration, thus enhancing anti-PD-L1 therapy response in urothelial and colorectal cancers [152,153]. In addition to affecting T cells, TGFβ signaling in the TME can also decrease NK cell activation and cytotoxic activity via induction of miR-183, which reduces the expression of the NK-activating receptor NKp30 [154]. Hence, targeting the activity of CAFs can boost the anti-tumorigenic immune responses, and a combination of such a strategy with immunotherapy bears promises for enhanced treatment outcomes.

### 3.8. Targeting CAFs as an Anti-Cancer Therapeutic Approach

Patients with metastatic cancers frequently relapse and experience tumor recurrence despite the progress made in targeting cancer and the availability of different treatment strategies. In these cases, it may be enough that a few cancer cells or colonies are able to evade apoptosis and sustain their survival programs upon exposure to the treatment and gradually become reprogramed for tissue re-colonization [155]. Such cells can gain mechanisms to re-grow massively, while not responding to further drug treatment, or eventually acquiring broader drug resistance via processes also regulated by the TME [155]. Within the TME, CAFs have emerged as important regulators of cancer cell survival and resistance to therapies. For instance, in breast and ovarian cancers, elevated stromal signatures correlate with resistance to chemotherapeutic treatment [156].

The effect of CAFs in mediating anti-cancer drug resistance can occur through the modulation of pathways involved in the ECM-cancer cell interactions, paracrine signaling, or even via direct CAF-cancer cell contact [157]. An example of such resistance-driving ECM interaction is the increased resistance to BRAF inhibitors in melanoma, which occurs when CAFs generate a fibronectin-rich stiff TME that leads to enhanced cancer cell survival via the fibronectin-activated β1-integrin-FAK-ERK axis [158]. Regarding the CAF-dependent paracrine signals, MMPs secreted by CAFs enhance the resistance of head and neck cancer cells to anti-epithelial growth factor receptor targeted treatments [159]. The CAF secretome can also directly activate pro-survival signaling cascades in tumor cells upon exposure to drug treatment. For example, Wnt signaling is triggered in cancer cells due to the secretion of WNT16B and secreted frizzled-related protein 2 ligand by CAFs, eventually attenuating the effect of cytotoxic drugs in prostate cancer cells in vitro and in vivo [160,161]. Moreover, IL-6 secreted by CAFs increases cancer cell survival and resistance to tamoxifen treatment in luminal breast cancer [162]. In response to PDGF-CC secreted by breast cancer cells, the CAFs can in turn start to express HGF, insulin-like growth factor-binding protein 3 and stanniocalcin 1, which also induce the acquisition of tamoxifen resistance and basal-like phenotype in cancer cells [163]. Recently, a subset of CAFs expressing membrane metalloendopeptidase and G protein-coupled receptor 77 has been identified in breast cancer patients, which sustain cancer stemness and correlate with poor prognosis, as well as resistance to chemotherapy [164].

As discussed above, CAFs can also decrease the efficacy of immunotherapy. For example, CXCL12 expressing CAFs reduce the effect of anti-CTLA-4 and PD-L1 antagonists in PDAC tumor cells [165]. When targeting the CXCL12-CXCR4 signaling pathway, cytotoxic T cells are rapidly recruited and form a potent anti-tumorigenic microenvironment, diminishing the PDAC cell growth [165]. Targeting CAFs in the TME is, therefore, an attractive strategy to consider and test. The anti-stromal drugs may offer new combinatorial strategies to overcome the drug resistance drawback. However, more systematic and comprehensive studies are required to identify the specific targetable-signaling cascades in CAFs and their cancer-associated ECM within a specific TME.

## 4. Conclusions

The anti-tumorigenic functions of normal fibroblasts are becoming evident based on the extensive findings highlighted in the current review. Normal fibroblasts can inhibit cancer cell growth and development via direct cell-cell interactions and secreted paracrine factors, as well as by maintaining normal tissue architecture through suppressive ECM structures and adhesion-related signaling cascades. Nonetheless, the incomplete understanding of the exact molecular mechanisms behind such actions introduces several challenges, and will require further research to address the clinical significance and utility of these observations. Specifically, increased understanding of the possibilities to restore the fibroblast-dependent growth-suppressive tissue integrity in the cancerous microenvironment could open new windows for cancer therapies. On the other hand, the pro-tumorigenic activity of CAFs has been studied extensively, as evidenced by more than 7500 published scientific articles on this topic. The majority of these studies highlight the significance of CAF-cancer cell crosstalk in tumor growth, invasion, and metastasis, as well as the CAF-induced cancer cell resistance against different anti-cancer drugs and treatments. Hence, combinational therapies that target CAFs and cancer cells simultaneously could prove useful. However, CAF heterogeneity is a challenge for such approaches: The absence of specific markers to identify CAFs implies the existence of several subsets, which may reflect their variability regarding the phenotypic state as well as the cell or tissue of origin, and thereby also the signaling mediators and mechanisms to target. In conclusion, more systematic and comprehensive studies are required to stratify the heterogeneity of CAFs and investigate the possibility to halt cancer progression by inducing cross switches between the different fibroblast phenotypes.
